# Oxidative Stress Biomarkers in Silicosis: A Systematic Review

**DOI:** 10.3390/diseases14030098

**Published:** 2026-03-06

**Authors:** Maria Carmina Pau, Elisabetta Zinellu, Barbara Piras, Alice Nardi, Maria Roberta Lacana, Chiara Scala, Angelo Zinellu, Arduino A. Mangoni, Ciriaco Carru, Alessandro G. Fois, Gaetano Caramori, Pietro Pirina

**Affiliations:** 1 Clinical and Interventional Pulmonology, University Hospital of Sassari (AOU), 07100 Sassari, Italybarbara.piras@aouss.it (B.P.); agfois@uniss.it (A.G.F.); 2 Department of Medicine, Surgery and Pharmacy, University of Sassari, 07100 Sassari, Italy; a.nardi@studenti.uniss.it; 3 Department of Biomedical Sciences, University of Sassari, 07100 Sassari, Italy; azinellu@uniss.it (A.Z.); carru@uniss.it (C.C.); 4 Discipline of Clinical Pharmacology, College of Medicine and Public Health, Flinders University, Bedford Park, SA 5042, Australia; arduino.mangoni@flinders.edu.au; 5 Department of Clinical Pharmacology, Flinders Medical Centre, Southern Adelaide Local Health Network, Bedford Park, SA 5042, Australia; 6 Pulmonology, Department of Medicine and Surgery, University of Parma, 43125 Parma, Italy; gaetano.caramori@unipr.it

**Keywords:** silicosis, pneumoconiosis, oxidative stress, lipid peroxidation, ROS

## Abstract

Background: Silicosis is a progressive fibrotic lung disease caused by chronic inhalation of crystalline silica. Increasing evidence indicates that oxidative stress plays a central role in linking silica exposure to inflammation, tissue injury, and fibrosis. We conducted a systematic review to critically appraise the current evidence on the imbalance between oxidant and antioxidant markers in patients with silicosis compared with unexposed healthy controls. Methods: A systematic literature search was conducted in PubMed, Scopus, and Google Scholar from their inception to 30 November 2025. Eligible studies assessed oxidative stress biomarkers in biological samples from patients with silicosis and non-exposed controls. Results: Malondialdehyde (MDA) and Superoxide Dismutase (SOD) were the most frequently assessed oxidative and antioxidant markers, respectively, with MDA significantly increased and SOD decreased in patients with silicosis, highlighting amplified lipid peroxidation and impaired antioxidant defense. In addition, elevated levels of other oxidant molecules confirmed the presence of lipid, nitrosative, and DNA oxidative damage. Overall, antioxidant defenses were compromised, although some markers appeared to vary with disease stage. Conclusions: This review highlights the central role of oxidative stress in the pathogenesis and progression of silicosis. Future studies with larger cohorts and a broader range of biomarkers are needed to better understand oxidative imbalance and its potential utility for monitoring disease progression and assessing severity in this population.

## 1. Introduction

Silicosis is a fibrotic lung disease caused by prolonged exposure to crystalline silica dust, primarily affecting workers in mining, sandblasting, and quarrying industries [1,2]. The disease remains a significant public health concern in these occupational settings, despite the implementation of preventive measures [3,4].

The histopathological hallmark of silicosis is well established as progressive pulmonary fibrosis; nevertheless, the full spectrum of molecular and cellular events involved in fibrogenesis following silica exposure is highly complex and remains the subject of extensive investigation [5,6].

Among the mechanisms proposed for silica-induced lung injury, oxidative stress is increasingly recognized as a key mediator linking initial particle-lung cell interactions to the development of persistent inflammation and resulting fibrosis [6,7,8].

After inhalation, crystalline silica particles are phagocytosed by alveolar macrophages, which triggers the production of reactive oxygen species (ROS) and pro-inflammatory cytokines, as well as activation of the NLRP3 inflammasome [9]. The resulting oxidative stress contributes to lipid, DNA, and protein damage, promoting tissue injury [10].

Injured macrophages induce the release of cytokines and Damage-Associated Molecular Patterns (DAMPs), promoting the recruitment of various immune cells, while ROS activate intracellular pathways, thereby enhancing cytokine expression [6,11]. These signaling pathways also favor epithelial–mesenchymal transition and stimulate fibroblast proliferation, contributing to fibrotic remodeling and alterations in lung structure [12,13].

**Figure 1:**
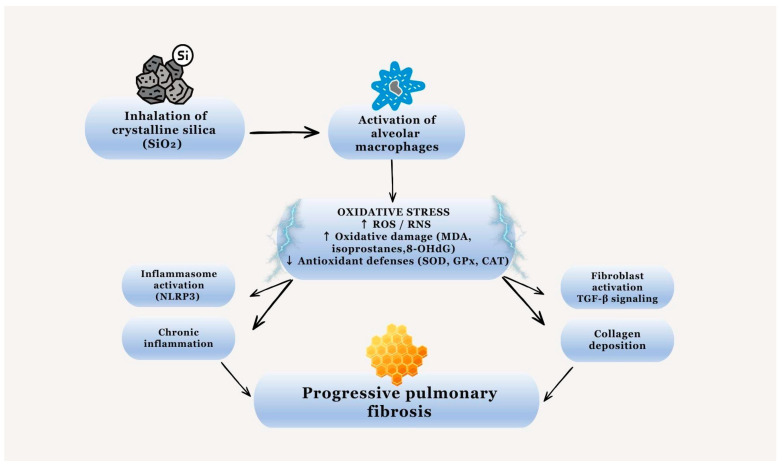
Schematic representation of the main pathogenetic mechanisms involved in silicosis. Inhaled crystalline silica particles are phagocytosed by alveolar macrophages, leading to excessive production of reactive oxygen and nitrogen species (ROS/RNS), oxidative damage, inflammasome activation, and persistent inflammation. The imbalance between oxidant and antioxidant systems promotes fibroblast activation and progressive pulmonary fibrosis.

The repeated cycle of silica phagocytosis by macrophages, cell damage, and particle release leads to sustained and further increased ROS production, as well as activation of the inflammatory response, thereby contributing to the progression toward chronic fibrosis [14,15]. The main pathogenetic mechanisms involved in silicosis are described in Figure 1.

Several studies in animal models, particularly rats, have investigated the effects of crystalline silica exposure on oxidative stress and antioxidant defenses, providing mechanistic support for the role of these processes in silica-induced lung injury [16,17,18]. These models, based on intratracheal instillation of, or inhalation exposure to, respirable crystalline silica, have demonstrated early and sustained ROS overproduction, increased lipid peroxidation, and impairment of endogenous antioxidant defenses in lung tissue [16,19]. In addition to enhanced superoxide and hydroxyl radical production, silica exposure has been shown to induce mitochondrial dysfunction in alveolar macrophages and epithelial cells. This includes loss of mitochondrial membrane potential and higher mitochondrial ROS production, which further increases intracellular oxidative stress and redox imbalance [17,20]. Moreover, animal studies have shown that oxidative stress precedes and amplifies inflammatory cell recruitment and fibroblast activation, ultimately contributing to collagen deposition and progressive pulmonary fibrosis [21,22]. These preclinical models have therefore provided crucial mechanistic insight into the causal relationship between silica exposure, redox imbalance, and fibrogenesis.

Supporting evidence in human-derived cellular models also confirms the significant oxidative potential of crystalline silica. For instance, exposure of human bronchial epithelial cells (BEAS-2B) to crystalline silica Min-U-Sil 5 caused early and extensive ROS generation, accompanied by a reduction in key intracellular antioxidant and antiglycation defenses, highlighting the important role of oxidative stress in silica-induced cellular injury [23]. In addition, exposure to silica has been shown to activate the redox-sensitive signaling pathways, such as NF-κB and MAPKs, leading to upregulation of pro-inflammatory mediators, including IL-6 and IL-8, which contribute to the initiation of inflammatory responses relevant to pulmonary injury [24,25].

Consistent with findings in human-derived cell models, oxidative stress has also been documented in clinical settings, with studies examining both patients with clinically manifest disease and workers occupationally exposed to silica. These studies reported high levels of malondialdehyde (MDA) and reduced activity of key antioxidant enzymes, including superoxide dismutase (SOD), catalase (CAT), and glutathione peroxidase (GPx) in patients with silicosis [26,27,28,29].

In the literature, the groups most often compared with respect to oxidative stress markers are patients with silicosis and workers exposed to silica [28,30,31]. Given that both groups are exposed, differences in oxidative alterations may be diluted by ongoing biological responses in both cohorts. Conversely, comparing non-exposed subjects with patients who have developed the disease could provide a more robust assessment of such alterations. This approach would allow for a clearer identification of specific biomarkers that distinguish pathological fibrosis from general occupational exposure, thereby highlighting the cumulative impact of crystalline silica on systemic redox homeostasis.

Therefore, this systematic review sought to critically appraise the available evidence on oxidative stress biomarkers in silicosis, focusing on comparisons between patients with silicosis and unexposed healthy controls. This approach aims to define a true redox baseline and identify clinically relevant biomarkers for understanding pathogenesis and disease progression.

## 2. Materials and Methods

### Search Strategy and Study Selection

A systematic literature search was conducted across the electronic databases PubMed, Scopus, and Google Scholar, from inception to 30 November 2025. The search strategy used the following terms: “oxidative stress” AND “silicosis” OR “oxidative stress” AND “pneumoconiosis”. No additional filters or limits were applied.

After retrieving records from the three databases, duplicates were identified manually by comparing titles, authors, and publication years, and subsequently removed. The relevance of the remaining records was evaluated manually based on their titles and abstracts. Given the large number of results retrieved from Google Scholar, only records deemed potentially relevant based on title screening were considered. Specifically, records were considered “not relevant” if they clearly did not address oxidative stress or silicosis/pneumoconiosis, were conducted in non-human populations, or were non-original documents such as theses, conference abstracts, citations, or narrative reviews. After removing duplicates across databases, records were screened by title and abstract using predefined inclusion and exclusion criteria.

The inclusion criteria were: (i) assessment of oxidative stress biomarkers in any biological specimen; (ii) comparison between patients with silicosis and healthy controls unexposed to silica; (iii) publication in the English language; and (iv) availability of the full text. Studies without comparison between patients with silicosis and healthy controls or without the availability of the full text and non-English publications were excluded. Data extraction was performed independently by two reviewers. Any discrepancies were resolved through discussion, and when necessary, a third reviewer was consulted. Full-text articles were assessed for eligibility. No automation tools were used, and no contact with study investigators was required to obtain or verify data. This study was conducted and reported in full accordance with the Preferred Reporting Items for Systematic Reviews and Meta-Analyses (PRISMA) 2020 statement and was not registered in the PROSPERO database (Table S1).

**Figure 2:**
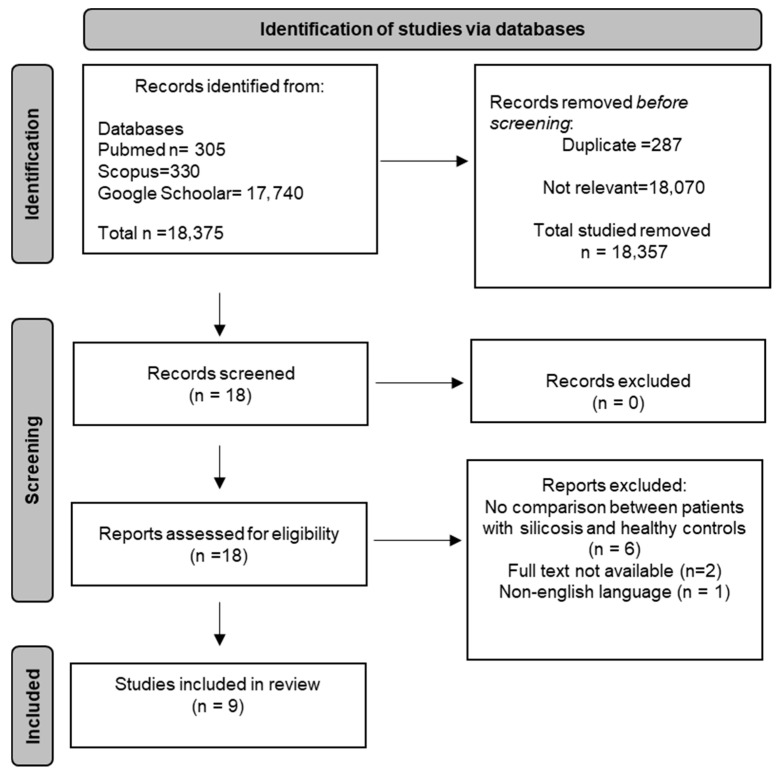
PRISMA 2020 flow diagram.

A flowchart illustrating the study selection process is presented in Figure 2.

## 3. Oxidative Stress Biomarkers in Silicosis Patients

### 3.1. Brief Overview

#### 3.1.1. Study Population

A total of nine studies were included in the present review, comprising 650 patients with silicosis (mean age 51 years, 87% males) and 383 unexposed controls (mean age 50 years, 94% males). The selected studies investigated a range of oxidative and antioxidant biomarkers, which are summarized in Table 1. The main demographic characteristics of patients and controls, as well as smoking status, are reported in Table 2.

#### 3.1.2. Oxidative Stress Biomarkers

Regarding the oxidant markers, malondialdehyde (MDA), a commonly used indicator of lipid peroxidation, was the most frequently assessed. A total of five studies measured free MDA directly; meanwhile, thiobarbituric acid reactive substances (TBARS), a broader assay measuring MDA, were reported in one study.

A further indicator of lipid peroxidation, F2-isoprostane, produced through the non-enzymatic oxidation of arachidonic acid, was quantified in three studies. In one of these, the parameter was specifically measured as 8-iso-prostaglandin F2.

Regarding oxidative damage to nucleic acids, hydroxy-2-deoxyguanosine (8-OHdG) was measured in two studies.

In addition to lipid peroxidation products and DNA oxidation markers, only one study assessed reactive nitrogen species. Specifically, nitric oxide (NO) and nitric oxide synthase (NOS) were measured as indicators of nitrosative stress.

In the included studies, the antioxidant defense system was also evaluated, investigating both enzymatic and non-enzymatic components.

Among antioxidant enzymes, superoxide dismutase (SOD), which detoxifies superoxide radicals, was the most frequently investigated marker across five studies.

Glutathione peroxidase (GPx), which reduces hydrogen peroxide and lipid peroxides using reduced glutathione (GSH), was investigated across four studies. Glutathione reductase (GR), which regenerates GSH from its oxidized form (GSSG), was evaluated in two studies. Finally, catalase, another enzyme involved in hydrogen peroxide detoxification, was assessed in a single study.

Among non-enzymatic antioxidants, only one study directly assessed GSH levels. Moreover, in one study, vitamin C (ascorbic acid), a key water-soluble antioxidant that neutralizes reactive oxygen species, was quantified.

In addition to individual enzymatic and non-enzymatic antioxidants, total antioxidant capacity (TAOC) was assessed in one study as a global indicator of the body’s overall defense against oxidative stress.

#### 3.1.3. Biological Matrices

Serum was the primary biological matrix for oxidative stress in six studies, specifically lipid peroxidation markers (MDA, TBARS), oxidative DNA damage (8-OHdG), and reactive nitrogen species (NO and NOS), as well as all antioxidant parameters, including enzymatic antioxidants (SOD, GPx, GR, and catalase), non-enzymatic antioxidants (GSH and vitamin C), and total antioxidant capacity (TAOC).

Plasma was used in three studies, primarily for the evaluation of lipid peroxidation markers, including MDA and F2-isoprostanes (including 8-iso-prostaglandin F2α).

Urine was employed in two studies to assess lipid peroxidation markers, specifically MDA and F2-isoprostanes.

Finally, exhaled breath condensate (EBC) was used in two studies to assess lipid peroxidation markers, particularly MDA and F2-isoprostanes, providing information on lung-specific oxidative injury.

#### 3.1.4. Quantification Methods

Different analytical methods were employed depending on the biomarker assessed. HPLC was used in one study to measure MDA, providing direct quantification of lipid peroxidation. Spectrophotometric assays were used in three studies: two quantified MDA, and one measured enzymatic antioxidant, including SOD and GPx. ELISA was used in four studies, for MDA in one study, enzymatic antioxidants (SOD, GPx, GR, catalase) in two studies, and non-enzymatic antioxidants such as GSH in one study. LC–ESI–MS/MS was employed in two studies for lipid peroxidation markers, including MDA, F2-isoprostanes/8-iso-prostaglandin F2α. Finally, standard biochemical or colorimetric assays were used in two studies, one assessing reactive nitrogen species (NO and NOS) and one measuring total antioxidant capacity (TAOC).

### 3.2. Oxidant Biomarkers

Across the included studies, oxidant markers were consistently elevated in patients with silicosis, suggesting a link between elevated oxidative stress and tissue injury. A detailed quantitative overview of oxidant biomarkers across different biological matrices (serum, plasma, urine, and exhaled breath condensate) is provided in Table 3. Overall, MDA was consistently increased in silicosis patients, confirming enhanced systemic lipid peroxidation.

The most frequently reported oxidant marker was MDA, which is used to assess lipid peroxidation. MDA levels were found to be significantly elevated in the serum of patients with silicosis compared with healthy controls, as demonstrated by Miao et al. [28], He et al. [36]. A similar trend was observed in the study by Nardi et al. [31], who quantified MDA levels in plasma samples, and Asku et al. [34], who measured MDA in both plasma and urine. In line with these findings, He et al. [36] also reported a significant, stage-dependent increase in MDA levels in patients with silicosis, with values consistently higher than those observed in healthy controls.

Peclová et al. (2011) [35] measured MDA in three types of biological samples—plasma, urine, and exhaled breath condensate (EBC). They reported a significant increase in MDA in the plasma of silicosis patients, whereas in urine and EBC, no significant differences were observed between patients and healthy controls.

Meanwhile, Scalia et al. [27] did not measure MDA specifically but quantified TBARS—a less specific spectrophotometric assay reflecting MDA and related aldehydes—and found these levels elevated in the presence of silicosis.

These studies used different analytical techniques to quantify lipid peroxidation: Nardi et al. [31] measured MDA using HPLC, Miao et al. [21,28] and He et al. [36] used a spectrophotometric assay, Peclová et al. [35] applied Liquid Chromatography–Electrospray Ionization–Tandem Mass Spectrometry (LC–ESI–MS/MS), and Asku et al. [34] measured MDA through Enzyme-Linked Immunosorbent Assay (ELISA).

Despite methodological heterogeneity, all studies consistently reported elevated MDA levels in patients with silicosis compared with non-exposed controls, indicating a marked increase in lipid peroxidation and emphasizing systemic oxidative stress and its influence on tissue damage.

Regarding lipid peroxidation, Peclová et al. [37] reported a significantly high level of 8-isoprostane, an oxidized derivative of arachidonic acid, in EBC samples of patients with silicosis. This result was confirmed in the study by Peclová et al. [35] using the same methodological system (LC-ESI-MS/MS); however, in the same study, urine and plasma samples showed higher levels of 8-isoprostane in 37 silicosis patients compared with 30 controls, although the differences were not statistically significant, unlike the significant increases observed in patients with asbestosis. On the contrary, in the study by Asku et al. [34], positive correlations were observed between silicosis status and F2-isoprostane levels in both urine and plasma, measured by ELISA. In addition to MDA and isoprostanes, other secondary products of lipid peroxidation were also investigated. Overall, MDA together with F2-isoprostanes, reinforces the evidence that silica exposure is associated with widespread oxidative membrane damage, affecting both circulating compartments and lung-derived samples.

In addition, two studies showed that the oxidative damage is not limited to lipids. In fact, both Ergün et al., 2025 and Ergün et al., 2023 [32,33] reported significantly higher 8-OH-dG levels in silicosis patients compared with controls, highlighting oxidative DNA injury in silica-exposed individuals.

Finally, Miao et al. [28] analyzed indicators of nitrosative stress. NO levels in serum were significantly higher in 130 patients with silicosis compared with 100 healthy controls. NOS, the enzyme responsible for NO production, was also elevated, confirming the marked increase in nitrosative stress in silicosis and reflecting enhanced enzymatic activity that contributes to NO overproduction. Nevertheless, in this study, no significant differences in these parameters were observed across different stages of silicosis. In contrast, NO levels in the study by Scalia et al. [27] did not differ between 31 non-exposed subjects and 37 patients with silicosis; however, NO was instead influenced by smoking status rather than by silica exposure or disease severity.

### 3.3. Antioxidant Biomarkers

In general, the capacity of the oxidant system in patients with silicosis appears compromised in the analyzed studies. A comprehensive summary of antioxidant biomarker alterations, including enzymatic and non-enzymatic defenses, is presented in Table 4. Overall, most studies reported reduced antioxidant capacity in silicosis patients.

The activity of SOD, the first-line defense enzyme, is generally reported as significantly reduced in the serum of silicosis patients in comparison with the control group [27,28,32,33]. An exception was the study by He et al. [36], in which SOD activity was significantly elevated in 128 patients with silicosis compared with 30 control subjects; however, its levels progressively decreased with increasing disease duration. The SOD marker was assayed using ELISA test [28,32,33] and spectrophotometric methods [27,36].

Similarly, GSH-Px activity is consistently found to be significantly lower in all three studies [28,32,33], severely impairing the detoxification of peroxides.

In particular, in the two studies by Ergün et al. [32,33], GPx levels were evaluated at both early and advanced stages of silicosis, with the biomarker being lower in the early stage. This may suggest that antioxidant defense mechanisms increase as the disease becomes more severe, as a response to oxidative stress. In addition, these studies [32,33] showed an increase in serum GR in patients with silicosis in comparison with non-exposed silica controls and a decrease in serum levels of catalase in the same groups, although these differences were not significant. These enzymatic patterns suggest a non-uniform antioxidant response, with selective vulnerability of specific defense mechanisms.

Even non-enzymatic defenses appear to be depleted, with Nardi et al. [31] demonstrating a significant reduction in plasma Vitamin C levels in 24 individuals with silicosis compared with 30 occupationally unexposed silica workers. This could reflect accelerated antioxidant consumption secondary to persistent oxidative stress induced by silica exposure. Meanwhile, serum GSH levels were observed to be increased in patients with silicosis compared with controls in the study by He et al. [36], but decreased as silicosis severity progressed.

Finally, total antioxidant capacity (TAOC) was measured in the study groups by Miao et al. [28]. Its levels were, as expected, significantly higher in the control group. Taken together, the antioxidant biomarker profile presented in Table 4 supports the presence of impaired redox homeostasis in silicosis, characterized by reduced antioxidant defenses and increased oxidative damage.

## 4. Discussion

This systematic review provides a comprehensive assessment of oxidative stress and associated biomarkers in silicosis. Overall, studies have indicated an imbalance between oxidant and antioxidant systems in patients affected by silicosis, highlighting how oxidative stress may play an important role in the progression of silicosis-induced lung damage.

The pathogenicity of silicosis is induced by the inhalation of silica particles, which activate macrophages, resulting in cellular damage, activation of cytokines and the inflammatory cascade, and oxidative stress caused by the formation of ROS. The process leads to pulmonary fibrosis, a consequence of sustained fibroblast activation and excessive collagen deposition in the lungs [5,38].

In addition to the overproduction of ROS, scientific evidence indicates that silica exposure is capable of activating the NLRP3 inflammasome through ROS-dependent signaling pathways, promoting the release of IL-1β and the amplification of the inflammatory cascade [39]. Moreover, mitochondrial dysfunction and altered ATP metabolism in macrophages have been linked to persistent oxidative stress and fibroblast activation [40]. In this context, oxidative stress is not merely a secondary consequence of the inflammatory process, but rather a central factor underlying epithelial injury and the progressive deposition of the extracellular matrix. A persistent redox imbalance may therefore promote TGF-β-mediated myofibroblast differentiation and fibrotic progression, further strengthening the link between oxidative pathways and structural lung remodeling [41].

Several biomarkers were investigated in the studies analyzed. Among them, SOD, which represents the first line of defense against oxidative stress, was the most common antioxidant marker measured in the study population and was mainly found to be significantly decreased in patients with silicosis. This confirms how SOD allows neutralization of the superoxide radicals produced in the lungs and systemic circulation [42].

Among oxidant biomarkers, MDA was the most frequently studied. MDA, as a product of lipid peroxidation, is a direct and reliable indicator of oxidative damage to cellular membranes and is particularly relevant in the lung [43]. In vivo studies in animal models have shown that prolonged exposure to silica dust leads to elevated lipid peroxidation, reflected by a significant increase in MDA levels in biological samples [44,45]. In particular, one study showed that this increase progresses with disease severity in patients with silicosis, from early stages to more advanced stages. In contrast, antioxidant enzymes such as GPx and SOD decreased compared with controls but showed no significant stage-dependent variation [28]. The stage-dependent increase in MDA may reflect cumulative lipid peroxidation damage; in the late stages, cellular damage and mitochondrial dysfunction may impair the regulation of antioxidant enzymes, indicating a dysregulated antioxidant response to persistent oxidative stress [16]. At the same time, the observed increase in GR may represent an attempt to regenerate reduced glutathione in response to continuous ROS production. In contrast, the absence of significant changes in catalase levels highlights a selective vulnerability within the antioxidant system. In other respiratory conditions, the antioxidant response to oxidative stress has been shown to be dynamic and non-uniform [46,47].

In the early stages of silica exposure, antioxidant enzymes may undergo transient upregulation as a compensatory response to increased ROS production. Conversely, during advanced disease stages, a prolonged oxidative burden can exhaust endogenous defense systems, leading to enzymatic depletion and the subsequent impairment of redox homeostasis. This dynamic imbalance may partially account for the discrepancies observed across studies and highlights the critical importance of accounting for disease staging when interpreting oxidative biomarkers [48,49].

In the reviewed studies, levels of F2-isoprostane were also elevated in the plasma and urine of silicosis patients, further highlighting an increased lipid peroxidation. However, no significant differences were observed in EBC, suggesting a possible tissue-specific variation or sampling limitations [35].

Overall, these analyses highlight the multifaceted nature of oxidative stress in silicosis, in which both enzymatic and non-enzymatic components are altered in a stage-dependent and tissue-specific manner. In addition to lipid peroxidation, the reviewed studies also report a significant increase in reactive nitrogen species and markers of oxidative DNA damage in patients with silicosis, further supporting the presence of widespread redox imbalance. This persistent imbalance between oxidants and antioxidants contributes not only to membrane lipid damage and genomic instability but also to mitochondrial dysfunction, chronic inflammation, and progressive fibrotic remodeling of the lung.

The oxidative profile observed in silicosis also shares similarities with other chronic inflammatory and fibrotic lung diseases. In idiopathic pulmonary fibrosis (IPF) and chronic obstructive pulmonary disease (COPD), persistent ROS generation contributes to epithelial injury, impaired antioxidant defenses, and progressive tissue remodeling [37,47,50]. However, in silicosis, the continuous retention of silica particles within the lungs provides a sustained pro-oxidant stimulus, explaining the marked lipid peroxidation and the stage-dependent increase in MDA levels observed in the reviewed studies. This suggests that oxidative stress in silicosis may be particularly persistent and particle-driven compared with other fibrotic lung disorders [47].

In addition, the assessment of multiple oxidative stress biomarkers across different biological matrices, which emphasizes the systemic nature of oxidative stress, provides a more comprehensive understanding of the impact of silica exposure and may allow the identification of potential targets for therapeutic interventions aimed at maintaining redox homeostasis. Moreover, demographic and clinical factors such as advanced age, comorbid metabolic or respiratory conditions, and cigarette smoking can influence oxidative and antioxidant biomarker levels, contributing to the discrepancies observed between studies and highlighting the importance of accounting for these confounding factors when evaluating the redox profile in patients with silicosis [51,52,53].

The main limitation of the present review is represented by the small number of studies addressing this issue in the specific groups of interest, particularly patients with silicosis compared with healthy, non-exposed subjects. The small number of studies included in the final analysis reflects both the predefined inclusion criteria applied and the current state of the literature on oxidative stress biomarkers in silicosis. However, these findings further highlight the need for more standardized, high-quality research in this field. In addition, the use of a wider range of biological matrices would allow a better understanding of the usefulness of specific oxidative stress markers depending on the type of sample analyzed. Furthermore, oxidative stress parameters may also be affected by demographic and lifestyle variables such as age, comorbidities, and cigarette smoking. Smoking, in particular, represents a well-established source of oxidative imbalance and may alter lipid peroxidation products and antioxidant defenses independently of pneumoconiosis. However, smoking status was inconsistently reported or adjusted for across the included investigations [27,28,31,32,33,34,35,36,37]. This heterogeneity may partially contribute to variability in biomarker levels between studies. Despite the limitations, the present study provides the first comprehensive review of the literature evaluating oxidative stress markers, including both oxidant and antioxidant molecules, in patients with silicosis compared with control subjects.

## 5. Conclusions

In conclusion, this systematic review highlights the central role of oxidative stress in the pathogenesis and progression of silicosis. Consistent evidence of increased lipid peroxidation and altered antioxidant defenses supports the idea that a persistent redox imbalance contributes to epithelial injury, inflammasome activation, and fibrotic remodeling. However, the heterogeneity observed across studies underscores the need for standardized methodologies, careful disease staging, and adequate control of confounding factors when interpreting oxidative biomarkers.

Further studies, including a larger number of investigations in which different types of oxidant and antioxidant molecules are measured, may provide additional information on oxidative imbalance and help clarify the role of oxidative stress markers in the pathogenesis, monitoring, and assessment of disease progression in silicosis using specific biomarkers. Moreover, dedicated studies comparing silica-exposed subjects who develop silicosis with similarly exposed individuals who remain disease-free will be essential to better clarify susceptibility factors, identify early biomarkers of vulnerability and explore potential redox-targeted therapeutic strategies.

## Figures and Tables

**Table 1:** Oxidative stress biomarkers in silicosis patients compared to unexposed silica controls.

Oxidative Stress Biomarkers	Biological Specimen	Silicosis Patients	Unexposed Controls	Ref.
8-OHdG; SOD; GR GPx; Catalase	Serum	71	52	[32]
8-OHdG; SOD; GR GPx; Catalase	Serum	116	55	[33]
TBARS-MDA SOD; NO	Serum	37	31	[27]
MDA F2 isoprostane	Plasma/Urine	47	30	[34]
MDA Vitamin C	Plasma Serum	24	30	[31]
MDA; SOD; GPx NO; NOS; TAOC	Serum	130	100	[28]
8-isoprostane MDA	Plasma Urine/EBC	37	30	[35]
MDA; SOD GSH	Serum	128	30	[36]
8-isoprostane	EBC	60	25	[37]

GSH: glutathione; GPx: glutathione peroxidase; GR: glutathione reductase; MDA: malondialdehyde; SOD: superoxide dismutase; NO: nitric oxide; NOS: nitric oxide synthase; TAOC: total antioxidant capacity; 8-OHdG: 8-hydroxy-20-deoxyguanosine; TBARS: thiobarbituric acid reactive substances.

**Table 2:** The demographic characteristics of silicosis patients and silica-unexposed controls of the included studies.

Ref.	Silicosis Patients				Unexposed Controls			
	N	Age Mean ± SD	Gender % Males	Smoking Status %	N	Age Mean ± SD	Gender % Males	Smoking Status %
[32]	71	42.5 ± 7.1	Not reported	Current: 59.2	52	40.9 ± 10.0	Not reported	Current:40.4
				Former: 25.4				Former: 15.4
				Never: 15.5				Never: 44.2
[33]	116	41.7 ± 7.1	100	Current: 59.5	55	41.3 ± 9.9	100	Current: 40
				Former: 24.1				Former: 16.4
				Never: 16.4				Never: 43.6
[27]	24	38.7 ± 5.8	100	Never: 70.8	12	39.5 ± 6.8	100	Never: 83.3
				Ever: 29.1				
	13	41.1 ± 5.6	100	Never: 46.2				Ever: 16.6
				Ever: 53.9				
[34]	47	41.8 ± 7.6	100	Smokers: 78.7	30	43.6 ± 9.6	100	Smokers: 43.3
[31]	24	55.7 ± 1.1	100	Smokers: 3.3	30	44.6 ± 2.1	100	Smokers: 8.3
				Former: 16.7				Former: 58.3
				Never: 76.7				Never: 29.2
[28]	130	52.0 ± 10.4	100	Not reported	100	48.7 ± 8.0	100	Not reported
[35]	37	69.1 ± 2.9	97	Smokers: 13.5	29	67.0 ± 4.6	69	Smokers: 13.8
[36]	128	61.3 ± 13.0	Not Reported	Smokers: 4.7	130	62.9 ± 12.3	Not Reported	Smokers: 7.7
[37]	60	66.9 ± 2.0	96	Smokers: 23.3	25	64.7 ± 4.8	92	Smokers: 32.0

**Table 3:** Oxidant biomarkers in silicosis patients compared to unexposed controls.

		Patients		Controls				
Biomarkers	Biological Specimen	N	Mean ± DS or Median (IQR)	N	Mean ± DS or Median (IQR)	*p* Value	Effect ↑ ↓	Ref.
MDA	EBC	37	17.3 ± 2.6 ng/mL	29	17.5 ± 2.0 ng/mL	n.s.	----	[35]
	Plasma	37	51.5 ± 5.3 ng/mL	29	43.2 ± 6.1 ng/mL	0.048	↑	[35]
	Urine	37	47 ± 1.2 ng/mmol Creat	29	41.0 ± 1.2 ng/mmol Creat	n.s	----	[35]
	Serum	128	8.6 ± 2.1 nmol/mL	130	4.1 ± 1.4 nmol/mL	<0.05	↑	[36]
	Serum	130	5.054 ± 1.204 μmol/L	100	4.027 ± 0.822 μmol/L	<0.05	↑	[28]
	Plasma	47	44.1 ± 14.6 nmol/mL	30	31.9 ± 10.5 nmol/mL	<0.05	↑	[34]
	Urine	47	30.2 ± 5.1 nmol/mL	30	25.2 ± 6.1 nmol/mL	<0.05	↑	[34]
	Plasma	24	9.35 ± 0.23 μmol/L	30	6.51 ± 0.28 μmol/L	<0.001	↑	[31]
	Plasma	37	Not reported	12	Not reported		↑	[27]
8-Isoprostane	EBC	60	73.6 ± 9.9 pg/mL	25	43 ±10 pg/mL	0.00007	↑	[37]
	EBC	37	88.4 ± 7.3 pg/mL	29	62.8 ± 8.9 pg/mL	<0.001	↑	[35]
	Plasma	37	54.9 ± 4.5 pg/mL	29	49.9 ± 6.3 pg/mL	n.s.	----	[35]
	Urine	37	42.52 ± 13.8 pg/mmol Creat	29	40.76 ± 20.6 pg/mmol Creat	n.s.	----	[35]
	Plasma	47	432.7 ± 88.8 pg/dL	30	321.9 ± 69.4	<0.05	↑	[34]
	Urine	47	450.0 ± 101.4 pg/dL	30	386.9 ± 112.7 pg/dL	<0.05	↑	[34]
8-OHdG	Serum	116	2615 (1780–3805) pg/mL	55	1505 (1280–1800) pg/mL	<0.001	↑	[33]
	Serum	71	2615 (1780–3805) pg/mL	52	1505 (1280–1800) pg/mL	<0.001	↑	[32]
NO	Serum	130	81.16 ± 35.17 µmol/L	100	45.62 ± 24.08 µmol/L	<0.01	↑	[28]
	Plasma	37	Not reported	12	Not reported	—	----	[27]
NOS	Serum	130	36.201 ± 7.782 µmol/L	100	33.812 ± 6.398 µmol/L	<0.05	↑	[28]

↑ increased levels; ↓ reduced levels; MDA: malondialdehyde; SOD: superoxide dismutase; NO: nitric oxide; NOS: nitric oxide synthase; 8-OHdG: 8-hydroxy-20-deoxyguanosine; Creat: creatinine.

**Table 4:** Antioxidant biomarkers in silicosis patients compared to unexposed controls.

		Silicosis Patients		Unexposed Controls				
Biomarkers	Biological Specimen	N	Mean ± DS or Median (IQR)	N	Mean ± DS or Median (IQR)	*p* Value	Effect (↑ ↓)	Ref.
SOD	Serum	128	102.1 ± 19 U/mL	130	72.6 ± 16.4 U/mL	<0.05	↑	[36]
	Serum	130	68.209 ± 21.528 U/mL	100	75.239 ± 24.02 U/mL	<0.01	↓	[28]
	Serum	116	0.04 (0.03–0.04) U/mL	55	2.02 (2.01–2.03) U/mL	<0.001	↓	[33]
	Serum	71	0.04 (0.03–0.05) U/mL	52	2.02 (2.01–2.03)	<0.001	↓	[32]
	Plasma	37	Not reported	12	Not reported	—	↓	[27]
GSH-Px	Serum	130	270.469 ± 39.228 U/mL	100	223.360 ± 46.838	<0.01	↑	[28]
	Serum	116	4.51 (3.65–5.65) nmol/min/mL	55	6.22 (5.38–6.43) nmol/min/mL	<0.001	↓	[33]
	Serum	71	4.51 (3.64–5.65) nmol/min/mL	52	6.2 (5.29–6.43) nmol/min/mL	<0.001	↓	[32]
GSH	Serum	128	11.7 ± 2.5 mg/L	130	4.4 ± 1.6 mg/L	<0.05	↑	[36]
T-AOC	Serum	130	13.048 ± 4.153 U/mL	100	11.639 ± 3.707 U/mL	<0.05	↑	[28]
Catalase	Serum	116	1.09 (0.58–1.60) U/mL	55	1.30 (0.67–1.52) U/mL	n.s	-----	[33]
	Serum	71	0.96 (0.48–1.34) U/mL	52	1.29 (0.67–1.55) U/mL	n.s.	-----	[32]
GSH reductase	Serum	116	4.33 (2.83–10) nmol/mL/min × 10^3^	55	4 (2.33–5.25) nmol/mL/min × 10^3^	n.s.		[33]
	Serum	71	4.33 (3.08–10) nmol/mL/min × 10^3^	52	4 (2.29–5) nmol/mL/min × 10^3^	0.033	↑	[32]
Vitamin C	Serum	24	7.62 ± 0.58 mg/L	30	4.44 ± 0.41 mg/L	<0.05	↑	[31]

↑ increased levels; ↓ reduced levels; GSH: glutathione; SOD: superoxide dismutase; T-AOC: total antioxidant capacity.

## Data Availability

No new data were created or analyzed in this study. All the data are presented in the original contributions included in this study. Further inquiries can be directed to the corresponding author.

## Supplementary Materials

The following supporting information can be downloaded at: https://www.mdpi.com/article/10.3390/diseases14030098/s1, Table S1: PRISMA 2020 Checklist [54].

## Abbreviations

The following abbreviations are used in this manuscript:

DAMPs	Damage-Associated Molecular Patterns
ROS	reactive oxygen species
GSH	glutathione
GPx	glutathione peroxidase
GR	glutathione reductase
MDA	malondialdehyde
SOD	superoxide dismutase
NO	Nitric Oxide
NO	Nitric Oxide Synthase
TAOC	Total Antioxidant Capacity
8-OHdG	8-hydroxy-20-deoxyguanosine
CAT	catalase
TBARS	thiobarbituric acid reactive substances
LC–ESI–MS/MS	Chromatography–Electrospray Ionization–Tandem Mass Spectrometry
ELISA	Enzyme-Linked Immunosorbent Assay

## References

[1] Barnes H., Goh N.S.L., Leong T.L., Hoy R. (2019). Silica-associated lung disease: An old-world exposure in modern industries. Respirology.

[2] Fazio J.C., Viragh K., Houlroyd J., Gandhi S.A. (2025). A review of silicosis and other silica-related diseases in the engineered stone countertop processing industry. J. Occup. Med. Toxicol..

[3] Hoy R.F., Jeebhay M.F., Cavalin C., Chen W., Cohen R.A., Fireman E., Go L.H.T., León-Jiménez A., Menéndez-Navarro A., Ribeiro M. (2022). Current globa lperspectives on silicosis-Convergence of old and newly emergent hazards. Respirology.

[4] Joshi S., Tiwari M., Pasi S., Kharkwal G. (2025). Global workplace health and safety policies for silicosis elimination and way forward. Discov. Public Health.

[5] Calabrese F., Montero-Fernandez M.A., Kern I., Pezzuto F., Lunardi F., Hofman P., Berezowska S., Attanoos R., Burke L., Mason P. (2024). The role of pathologists in the diagnosis of occupational lung diseases: An expert opinion of the European Society of Pathology Pulmonary Pathology Working Group. Virchows Arch..

[6] Yang B., Liu X., Peng C., Meng X., Jia Q. (2025). Silicosis: From pathogenesis to therapeutics. Front. Pharmacol..

[7] Adamcakova J., Mokra D. (2021). New Insights into Pathomechanisms and Treatment Possibilities for Lung Silicosis. Int. J. Mol. Sci..

[8] Makena P., Kikalova T., Prasad G.L., Baxter S.A. (2023). Oxidative Stress and Lung Fibrosis: Towards an Adverse Outcome Pathway. Int. J. Mol. Sci..

[9] Yin H., Fang L., Wang L., Xia Y., Tian J., Ma L., Zhang J., Li N., Li W., Yao S. (2022). Acute Silica Exposure Triggers Pulmonary Inflammation Through Macrophage Pyroptosis: An Experimental Simulation. Front. Immunol..

[10] Zhou H., Zhang Q., Liu C., Fan J., Huang W., Li N., Yang M., Wang H., Xie W., Kong H. (2024). NLRP3 inflammasome mediates abnormal epithelial regeneration and distal lung remodeling in silica-induced lung fibrosis. Int. J. Mol. Med..

[11] Tschopp J., Schroder K. (2010). NLRP3 inflammasome activation: The convergence of multiple signalling pathways on ROS production?. Nat. Rev. Immunol..

[12] Harijith A., Ebenezer D.L., Natarajan V. (2014). Reactive oxygen species at the crossroads of inflammasome and inflammation. Front. Physiol..

[13] Qu Y., Zhai R., Wang D., Wang Z., Hou G., Wu C., Tang M., Xiao X., Jiao J., Ba Y. (2023). Mitochondrial folate pathway regulates myofibroblast differentiation and silica-induced pulmonary fibrosis. J. Transl. Med..

[14] Du S.-L., Zhou Y.-T., Hu H.-J., Lin L., Zhang Z.-Q. (2024). Silica-induced ROS in alveolar macrophages and its role on the formation of pulmonary fibrosis via polarizing macrophages into M2 phenotype: A review. Toxicol. Mech. Methods.

[15] Hu H.J., Fu Y.Y., Du S.L., Zhang Y.H., Zhang Z.Q., Han G.Z. (2025). Role of macrophage ATP metabolism disorder in SiO_2_ induced pulmonary fibrosis: A review. Purinergic Signal..

[16] Guan Y., Li G., Liu N., Wang Y.H., Zhou Q., Chang M.Y., Zhao L.L., Yao S.Q. (2022). Expression of Thioredoxin System Protein Induced by Silica in Rat Lung Tissue. Biomed. Environ. Sci..

[17] Zhu Z., Yang G., Wang Y., Yang J., Gao A., Niu P., Tian L. (2013). Suppression of thioredoxin system contributes to silica-induced oxidative stress and pulmonary fibrogenesis in rats. Toxicol. Lett..

[18] Esfahani M., Rahbar A.H., Asl S.S., Bashirian S., Mir Moeini E.S., Mehri F. (2023). The Effects of Resveratrol on Silica-Induced Lung Oxidative Stress and Inflammation in Rat. Saf. Health Work..

[19] Liu N., Xue L., Guan Y., Li Q.Z., Cao F.Y., Pang S.L., Guan W.J. (2016). Expression of Peroxiredoxins and Pulmonary Surfactant Protein A Induced by Silica in Rat Lung Tissue. Biomed. Environ. Sci..

[20] Li S., Zhao J., Han G., Zhang X., Li N., Zhang Z. (2023). Silicon dioxide-induced endoplasmic reticulum stress of alveolar macrophages and its role on the formation of silicosis fibrosis: A review article. Toxicol. Res..

[21] Barbarin V., Nihoul A., Misson P., Arras M., Delos M., Leclercq I., Lison D., Huaux F. (2005). The role of pro- and anti-inflammatory responses in silica-induced lung fibrosis. Respir Res..

[22] Lam M., Tate M.D. (2025). Crystalline killer: The molecular cascade of silica toxicity from inflammation to fibrosis. Curr. Opin. Allergy Clin. Immunol..

[23] Antognelli C., Gambelunghe A., Del Buono C., Murgia N., Talesa V.N., Muzi G. (2009). Crystalline silica Min-U-Sil 5 induces oxidative stress in human bronchial epithelial cells BEAS-2B by reducing the efficiency of antiglycation and antioxidant enzymatic defenses. Chem. Biol. Interact..

[24] Peeters P.M., Perkins T.N., Wouters E.F., Mossman B.T., Reynaert N.L. (2013). Silica induces NLRP3 inflammasome activation in human lung epithelial cells. Part. Fibre Toxicol..

[25] Skuland T., Ovrevik J., Låg M., Schwarze P., Refsnes M. (2014). Silica nanoparticles induce cytokine responses in lung epithelial cells through activation of a p38/TACE/TGF-α/EGFR-pathway and NF-κΒ signalling. Toxicol. Appl. Pharmacol..

[26] Anlar H.G., Bacanli M., İritaş S., Bal C., Kurt T., Tutkun E., Hinc Yilmaz O., Basaran N. (2017). Effects of Occupational Silica Exposure on OXIDATIVE Stress and Immune System Parameters in Ceramic Workers in TURKEY. J. Toxicol. Environ. Health Part A.

[27] Scalia Carneiro A.P., Algranti E., Chérot-Kornobis N., Silva Bezerra F., Tibiriça Bon A.M., Felicidade Tomaz Braz N., Soares Souza D.M., de Paula Costa G., Bussacos M.A., de Paula Alves Bezerra O.M. (2020). Inflammatory and oxidative stress biomarkers induced by silica exposure in crystal craftsmen. Am. J. Ind. Med..

[28] Miao R.M., Zhang X.T., Guo P., He E.Q., Zhou F., Zhao D.K., Zhang Y.Y. (2012). Effect of oxidative stress on development of silicosis. World J. Respirol..

[29] Orman A., Kahraman A., Cakar H., Ellidokuz H., Serteser M. (2005). Plasma malondialdehyde and erythrocyte glutathione levels in workers with cement dust-exposure silicosis. Toxicology.

[30] Liu K., Sun L., Li S., Xu H. (2023). Combined application of multiple biomarkers for early auxiliary diagnosis of silicosis. Toxicol. Ind. Health.

[31] Nardi J., Nascimento S., Göethel G., Gauer B., Sauer E., Fão N., Cestonaro L., Peruzzi C., Souza J., Garcia S.C. (2018). Inflammatory and oxidative stress parameters as potential early biomarkers for silicosis. Clin. Chim. Acta.

[32] Ergün R., Ergün D., Özkan E., Bacanlı M., Onmaz D.C., Körez M.K. (2025). Evaluation of neopterin, oxidative stress, and immune system in silicosis. Turk. J. Biochem..

[33] Ergün R., Ergün D., Özkan E., Kurt O.K., Bacanli M., Körez M.K. (2023). Can Serum Chitotriosidase Levels, Immune, and Oxidative Stress Parameters Be Early Diagnostic Indicators in Patients with Silicosis?. J. Occup. Environ. Med..

[34] Aksu N., Samadi A., Yalçınkaya A., Çetin T., Eser B., Lay İ., Öziş T.N., Öztaş Y., Sabuncuoğlu S. (2020). Evaluation of oxysterol levels of patients with silicosis by LC- MS/MS method. Mol. Cell Biochem..

[35] Pelclová D., Fenclová Z., Syslová K., Vlčková S., Lebedová J., Pecha O., Běláček J., Navrátil T., Kuzma M., Kačer P. (2011). Oxidative stress markers in exhaled breath condensate in lung fibroses are not significantly affected by systemic diseases. Ind. Health.

[36] He J.-L., Zhang J.-W., Ly G.-C., Zhao Y., Hong X.-P. (2011). Study on the serum oxidative stress status in silicosis patients. Afr. J. Biotechnol..

[37] Pelclová D., Fenclová Z., Kacer P., Navrátil T., Kuzma M., Lebedová J.K., Klusácková P. (2007). 8-isoprostane and leukotrienes in exhaled breath condensate in Czech subjects with silicosis. Ind. Health.

[38] Liu T.T., Sun H.F., Han Y.X., Zhan Y., Jiang J.D. (2024). The role of inflammation in silicosis. Front. Pharmacol..

[39] Dostert C., Pétrilli V., Van Bruggen R., Steele C., Mossman B.T., Tschopp J. (2008). Innate immune activation through Nalp3 inflammasome sensing of asbestos and silica. Science.

[40] Bueno M., Lai Y.C., Romero Y., Brands J., St Croix C.M., Kamga C., Corey C., Herazo-Maya J.D., Sembrat J., Lee J.S. (2015). PINK1 deficiency impairs mitochondrial homeostasis and promotes lung fibrosis. J. Clin. Investig..

[41] Liu R.M., Desai L.P. (2015). Reciprocal regulation of TGF-β and reactive oxygen species: A perverse cycle for fibrosis. Redox Biol..

[42] Kinnula V.L., Crapo J.D. (2003). Superoxide dismutases in the lung and human lung diseases. Am. J. Respir. Crit. Care Med..

[43] Rahman I., Adcock I.M. (2006). Oxidative stress and redox regulation of lung inflammation in COPD. Eur. Respir. J..

[44] Gholami A., Golbabaei F., Teimori G., Kianmehr M., Yaseri M. (2019). Investigation of Blood and Urine Malondialdehyde Levels in Mice Exposed to Silica Dust. Open Biochem. J..

[45] Bagus Artana G.N.I., Artini G.A.I., Nyoman Arijana G.K., Ngurah Rai I.B., Indrayani A.V. (2022). Exposure Time of Silica Dust and the Incidence of Oxidative Stress, Inflammation, and Fibrosis in Rat Lungs. Maced. J. Med. Sci..

[46] Zinellu E., Zinellu A., Fois A.G., Carru C., Pirina P. (2016). Circulating biomarkers of oxidative stress in chronic obstructive pulmonary disease: A systematic review. Respir. Res..

[47] Albano G.D., Gagliardo R.P., Montalbano A.M., Profita M. (2022). Overview of the Mechanisms of Oxidative Stress: Impact in Inflammation of the Airway Diseases. Antioxidants.

[48] Kliment C.R., Oury T.D. (2010). Oxidative stress, extracellular matrix targets, and idiopathic pulmonary fibrosis. Free Radic. Biol. Med..

[49] Barnes P.J. (2022). Oxidative Stress in Chronic Obstructive Pulmonary Disease. Antioxidants.

[50] Fois A.G., Paliogiannis P., Sotgia S., Mangoni A.A., Zinellu E., Pirina P., Carru C., Zinellu A. (2018). Evaluation of oxidative stress biomarkers in idiopathic pulmonary fibrosis and therapeutic applications: A systematic review. Respir. Res..

[51] Vassalle C., Novembrino C., Maffei S., Sciarrino R., De Giuseppe R., Vigna L., de Liso F., Mercuri A., Bamonti F. (2011). Determinants of oxidative stress related to gender: Relevance of age and smoking habit. Clin. Chem. Lab. Med..

[52] Zinellu E., Zinellu A., Fois A.G., Pau M.C., Scano V., Piras B., Carru C., Pirina P. (2021). Oxidative Stress Biomarkers in Chronic Obstructive Pulmonary Disease Exacerbations: A Systematic Review. Antioxidants.

[53] Salem AATrares K., Kohl M., Jansen E., Brenner H., Schöttker B. (2022). Long-term effects of smoking on serum concentrations of oxidative stress biomarkers: Results of a large, population-based cohort study. Environ. Res..

[54] Page M.J., McKenzie J.E., Bossuyt P.M., Boutron I., Hoffmann T.C., Mulrow C.D., Shamseer L., Tetzlaff J.M., Akl E.A., Brennan S.E. (2021). The PRISMA 2020 statement: An updated guideline for reporting systematic reviews. BMJ.

